# Antarctic krill oil exhibited synergistic effects with nobiletin and theanine in ameliorating memory and cognitive deficiency in SAMP8 mice: Applying the perspective of the sea–land combination to retard brain aging

**DOI:** 10.3389/fnagi.2022.964077

**Published:** 2022-09-16

**Authors:** Cheng-Cheng Wang, Jing-Ya Kong, Xiao-Yue Li, Jin-Yue Yang, Chang-Hu Xue, Teruyoshi Yanagita, Yu-Ming Wang

**Affiliations:** ^1^College of Food Science and Engineering, Ocean University of China, Qingdao, China; ^2^Laboratory for Marine Drugs and Bioproducts, Pilot National Laboratory for Marine Science and Technology, Qingdao, China; ^3^Laboratory of Nutrition Biochemistry, Department of Applied Biochemistry and Food Science, Saga University, Saga, Japan

**Keywords:** sea–land combination, neurodegenerative diseases, Antarctic krill oil, nobiletin, theanine, n-3 PUFAs-enriched phospholipids, Alzheimer's disease, neuroinflammation

## Abstract

The complex pathogenesis of Alzheimer's disease (AD) leads to a limited therapeutic effect; therefore, the combination of multiple bioactive ingredients may be more effective in improving AD due to synergistic effects. Based on the perspective of the sea–land combination, the effects of sea-derived Antarctic krill oil (AKO) combined with land-derived nobiletin (Nob) and L-theanine (The) on memory loss and cognitive deficiency were studied in senescence-accelerated prone 8 mice (SAMP8). The results demonstrated that AKO combined with The significantly increased the number of platform crossings in the Morris water maze test by 1.6-fold, and AKO combined with Nob significantly increased the preference index in a novel object recognition test. AKO exhibited synergistic effects with Nob and The in ameliorating recognition memory and spatial memory deficiency in SAMP8 mice, respectively. Further research of the mechanism indicated that AKO exhibited synergistic effects with Nob in suppressing β-amyloid (Aβ) aggregation, neurofibrillary tangles, and apoptosis and neuroinflammation, while the synergistic effects of AKO and The involved in synaptic plasticity and anti-neuroinflammation, which revealed that the combination was complex, not a mechanical addition. These findings revealed that the sea–land combination may be an effective strategy to treat and alleviate AD.

## Introduction

Aging is the primary risk factor for the development of most neurodegenerative diseases, including Alzheimer's disease (AD). AD is characterized by memory loss and cognitive impairment. Due to hidden symptoms, most patients with AD are difficult to diagnose and treat in time at the early stage of onset. In addition, the complex pathogenesis of AD leads to limited therapeutic effects, leading to irreversible development of AD and large socioeconomic and personal costs (Hou et al., 2019). Therefore, it is of great significance to develop a multitarget diet or natural bioactive ingredient to prevent the occurrence and development of AD. The Yin–Yang doctrine is an important scientific concept in modern biomedicine and nutrition, which is derived from ancient Chinese Philosophy (Sun et al., 2020). The opposite but complementary relationship between Yin and Yang is the key concept of the Yin/Yang doctrine, which is like that of the land and the sea. Foods from land and sea jointly contribute to human well-being with the coordination of resources and nutritional balance due to the complementary nutritional composition. Based on the Yin–Yang doctrine, it is considered that the sea–land combination may be an effective strategy to treat and alleviate AD.

Antarctic krill (*Euphausia superba*) oil (AKO) has been reported for its multiple health benefits as a novel food ingredient, which is abundant in phospholipids associated with n-3 polyunsaturated fatty acids (n-3 PUFAs), such as docosahexaenoic acid (DHA) and eicosapentaenoic acid (EPA) (Xie et al., 2019). A growing number of studies revealed that AKO significantly ameliorated memory impairment by inhibiting β-amyloid (Aβ) aggregation and its damage to neurons in mice (Li et al., 2018). In addition, previous studies suggested that n-3 PUFA-enriched phospholipids exhibited stronger effects than n-3 PUFA-enriched ethyl esters and triglyceride, and recombination of ethyl esters with phospholipids from egg yolk improved the dysfunction of memory and cognition in mice (Wang et al., 2019b).

It has been reported that the neuroprotective and anti-aging effects of phytochemicals derived from land, such as nobiletin (Nob, a representative lipophilic polymethoxylated flavone) and L-theanine (The, a unique hydrophilic amino acid of tea). Some evidence revealed that Nob could cross the blood–brain barrier (BBB) and enter the brain to inhibit neuronal damage in various animal models and cultured cells and slices (Braidy et al., 2017; Nakajima and Ohizumi, 2019). However, the oral bioavailability of Nob is limited due to its special chemical structure and limited solubility, which restricts its neuroprotective activity. Therefore, it was hypothesized that AKO phospholipids with amphiphilic and emulsifying performance had synergistic effects in ameliorating memory and cognitive deficiency with Nob by improving Nob bioavailability and multitarget effects. In addition, a large number of studies have reported that The significantly improves memory loss and cognitive deficiency by modulating neurotransmitter levels and inhibiting neuron loss, which may be a result of its glutamate-like chemical structure (Türközü and Sanlier, 2017). However, it was unclear whether the hydrophilic The had synergistic effects with the amphiphilic AKO in improving cognitive function.

Therefore, based on the proposed sea–land combination for coordinated resource development and balanced nutritional intake, the effects of the combination of sea-derived AKO and the land-derived phytochemicals (Nob and The) on memory loss and cognitive deficiency were invested in the present study in senescence-accelerated prone 8 mice (SAMP8). Furthermore, possible underlying molecular mechanisms were explored.

## Materials and methods

### Materials

Antarctic krill oil (purity >97%) was obtained from Kangjing Marine Biotechnology Co., Ltd. (Qingdao, China), and Nob (purity >98%) and The (purity >99%) were purchased from Solarbio Science & Technology Co., Ltd. (Beijing, China) and Jonk Biological Technology Co., Ltd. (Wuhan, China), respectively. The total DNA–RNA–Protein kit was purchased from Omega Bio-Tek, Inc. (San Francisco, USA). Primary antibodies were obtained from Cell Signaling Technology (Beverly, USA), ABclonal (Wuhan, China), or BIOSS (Beijing, China).

### Animals and treatments

Approximately 6-month-old male SAMP8 mice, SPF grade, were provided by the Animal Center of Medical College, Peking University (Beijing, China, SCXK(Jing)2016-0010), which were kept in a room with standard conditions and provided with food and water *ad libitum*. After adaptation, mice were randomly divided into six groups, including the model SAMP8 group, the AKO group, the Nob group, the The group, the combination of AKO and Nob groups (AKO + Nob), and the combination of AKO and The groups (AKO + The). Mice were fed a slightly modified AIN-93G standard diet consisting of 1% (w/w) AKO, 0.075% (w/w) Nob and The, correspondingly, for 3 months. Mice were evaluated by the Morris water maze test and novel object recognition test, followed by CO_2_ euthanasia with, and brains were rapidly separated and frozen with liquid nitrogen and then stored at −80°C until used or fixed in 4% buffered paraformaldehyde.

### Morris water maze test

The Morris water maze test was performed according to the previous protocol. In brief, after 5-day place navigation test, the 6th-day spatial probe test was carried out. Mice were monitored by a video camera on the apparatus. The time spent finding the platform during the place navigation test, and the number, time, and distance in the target quadrant, as well as the number of platform crossings in the spatial probe test, were recorded and analyzed using the ANY-maze software (Stoelting Co., Wood Dale, IL, USA).

### Novel object recognition test

In the familiarization phase of the novel object recognition test, mice were trained in a test chamber with two identical objects in a square open field. Briefly, mice were successively placed on the side of the wall away from the objects and back toward them. The time spent sniffing two objects was recorded. After 24 h, the test phase was performed. One of the objects in the test chamber was replaced with a novel one, and mice were monitored and recorded the time spent sniffing the familiar and the novel object. The preference index was calculated according to the following formula: preference index = time spent sniffing the novel object/(time spent sniffing the novel object + time spent sniffing the familiar).

### Bielschowsky silver staining, Nissl staining, and immunofluorescence

Fixed tissues were dehydrated and embedded in paraffin, followed by cutting into thin sections. Bielschowsky silver staining was performed by a modified method. Briefly, slices were immersed in 10% silver nitrate solution for 15 min and ammonium silver nitrate solution at 40°C for 30 min, and then stopped in 1% ammonium hydroxide solution. For Nissl staining, slices were stained with 0.5% cresyl violet, followed by dehydration with ethanol and xylene. To detect the levels of ionized calcium binding adapter molecule 1 (IBA1) and glial fibrillary acidic protein (GFAP) in the brain, slices were incubated with primary antibodies against IBA1 (1:400) and GFAP (1:400) and fluorescent-labeled secondary antibodies, respectively. Slices were viewed under a microscope and analyzed by image J.

### Protein extraction and western blotting assay

The total DNA–RNA–Protein kit was used to extract the total protein according to the manufacturer's instructions. The protein was separated by electrophoresis on 5–12% sodium dodecyl-sulfate polyacrylamide gel electrophoresis (SDS–PAGE) gels and transferred to polyvinylidene fluoride (PVDF) membrane. Blots were blocked in 5% bovine serum albumin (BSA) and then incubated with primary antibodies against amyloid precursor protein (APP, 1:2,000), β-site APP cleaving enzyme 1 (BACE1, 1:2,000), phosphorylated tau (p-Tau) (Ser396) (1:5,000), phosphorylated-glycogen synthase kinase-3β (Y216 + Y279) (p-GSK3β, 1:1,000), B-cell lymphoma 2 (Bcl-2, 1:1,000), Cleaved-Caspase-9 (1:1,000), Cleaved-Caspase-3 (1:2,000), synaptophysin (SYN, 1:5,000), postsynaptic density protein-95 (PSD-95, 1:1,000), brain-derived neurotrophic factor (BDNF, 1:2,000), Toll-like receptor 4 (TLR4, 1:2,000), tumor necrosis factor-α (TNF-α, 1:2,000), nuclear factor-κB (NF-κB, 1:2,000), NOD-like receptor family pyrin domain containing 3 (NLRP3, 1:2,000), and interleukin-6 (IL-6, 1:2,000) at 4°C overnight, respectively. After incubation with horse radish peroxidase-conjugated secondary antibodies (1:3,000) for 2 h, blots were visualized using an enhanced chemiluminescence (ECL, EpiZyme, China) substrate with a UVP Auto Chemi Image system (UVP, Inc., Upland, CA, USA). Protein load was evaluated using anti-β-actin antibodies (1:2,000, EpiZyme #LF201, China).

### Statistical analysis

All data were expressed as mean ± standard error of the mean (SEM, indicated by error bars), and significant differences were assessed by one-way analysis of variance (ANOVA) followed by a *post-hoc* test and Student's *t*-test. Latency curves in behavioral tests were analyzed with a two-way repeated-measures ANOVA (group × day) followed by a *post*-*hoc* test. Different letters indicate significant differences when *p* < 0.05.

## Results and discussion

### The effects of memory and cognitive deficiency on SAMP8 mice

**Figure 1:**
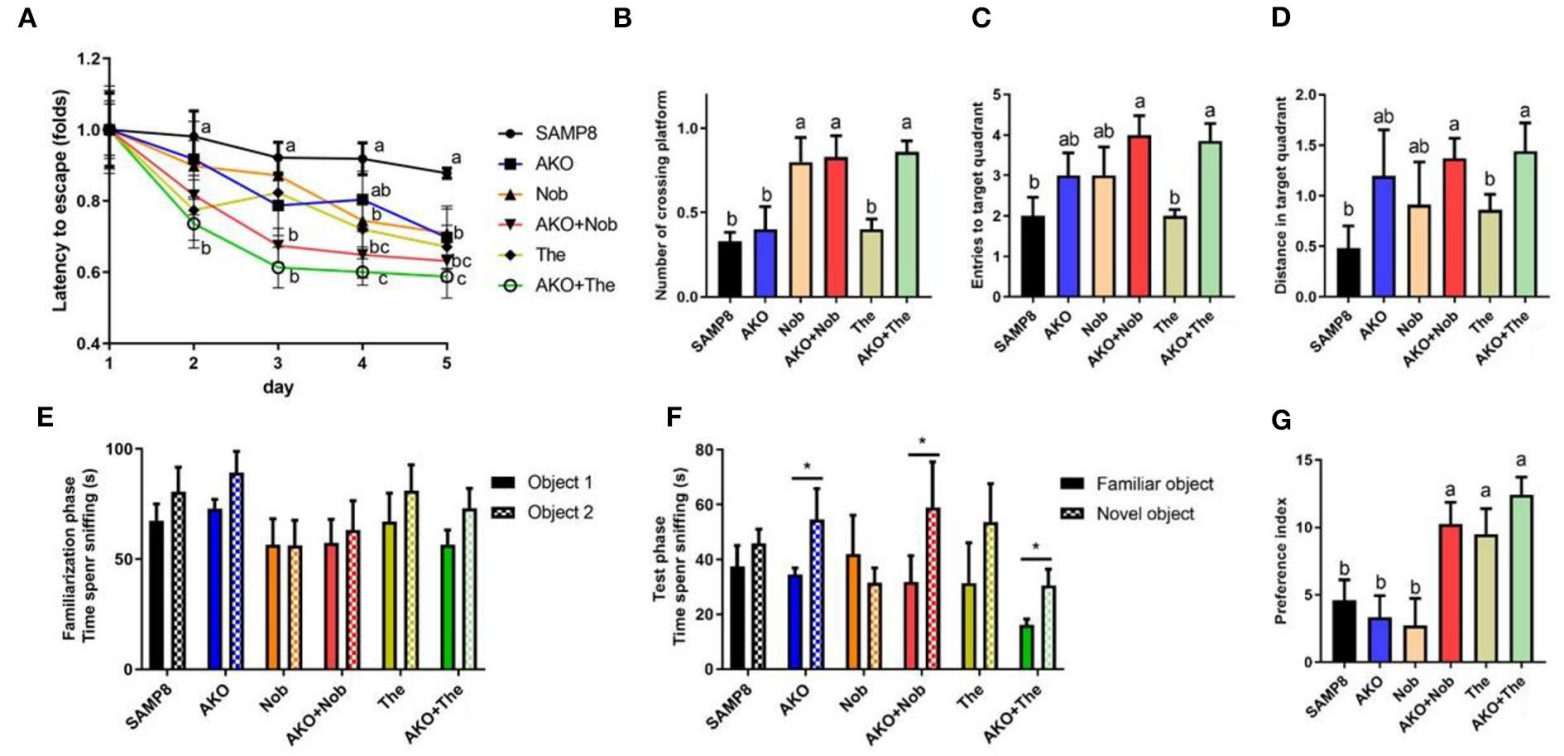
Effects on memory and cognitive deficiency indicated by performance in the Morris water maze test **(A–D)** and the novel object recognition test **(E–G)**. **(A)** Latency to escape to the platform during the place navigation test. **(B)** The number of platform crossings. **(C)** The entries to the target quadrant. **(D)** The distance traveled in the target quadrant. **(E)** The time spent sniffing two objects in the familiarization phase. **(F)** The time spent sniffing two objects in the test phase. **(G)** Increase of the preference index in two stages. All data were presented as mean ± standard error of the mean (SEM). Different letters indicate a significant difference at *p* < 0.05 by analysis of variance (ANOVA), and * indicates a significant difference at *p* < 0.05 by *t*-test.

The complex pathogenesis of AD leads to limited therapeutic effects; therefore, the development of multitarget bioactive ingredients to improve AD may be an effective strategy. In the present study, based on the Yin–Yang doctrine, the sea–land combination therapy strategy, sea-derived AKO combined with land-derived Nob or The, was attempted to improve memory and cognitive deficiency in SAMP8 mice. The Morris water maze test is a typical behavioral test to evaluate spatial memory and cognition of mice, which was performed in the present study. The results during the place navigation test suggested that the latency of SAMP8 mice decreased only slightly and not significantly after 5 days of the place navigation test, suggesting spatial memory and cognitive deficiency of SAMP8 mice. However, from the 2nd day until the 5th day, the latency of mice in the AKO+The group was significantly (*p* < 0.01) lower than that in the SAMP8 group and exhibited more superior effects than other diets. Dietary AKO+Nob significantly reduced latency from the 3rd day with no significant differences with AKO and Nob (Figure 1A). In the spatial probing test, the number of platform crossings, target quadrant entries, and target quadrant distance traveled were significantly increased with AKO+The though no significant difference was observed in AKO and The groups, compared with the SAMP8 group (Figures 1B–D). In addition, AKO+Nob exhibited significant effects by increasing the number of platform crossings (Figure 1B). The results from the Morris water maze test revealed that AKO exhibited synergistic effects with The rather than Nob in ameliorating spatial memory and cognitive deficiency in SAMP8 mice.

The novel object recognition test is a recognition memory test based on the innate preference of the rodent to explore the novel object rather than the familiar one. Mice remembered and preconized that the familiar object would spend more time exploring the novel object (Bevins and Besheer, 2006; Leger et al., 2013). The results suggested that no significant differences were observed between the time spent sniffing two objects in all groups during the familiarization phase. However, the time spent sniffing the novel object was significantly increased in the AKO, AKO+Nob, and AKO+The groups, compared with the familiar one during the test phase (Figures 1E,F). In addition, two-stage preference index enhancement was significantly increased with AKO+Nob, though no significant differences were observed in the AKO and Nob groups, compared with the SAMP8 group (Figure 1G), suggesting that AKO exhibited synergistic effects with Nob rather than The in ameliorating recognition memory in SAMP8 mice.

It has been reported that AKO significantly ameliorate memory impairment in mice and AKO-rich n-3 PUFA-enriched phospholipids exhibited more superior effects than n-3 PUFAs in other forms (Li et al., 2018; Wang et al., 2019b). In addition, Nob improved memory impairment and context-dependent fear memory impairment in SAMP8, and neuroprotective effects have been verified in other rodent models, such as Aβ-infused rats and 3XTg-AD mice (Onozuka et al., 2008; Nakajima et al., 2013; Ghasemi-Tarie et al., 2022b). Theanine significantly ameliorated D-galactose-induced brain damage in rats and Aβ-induced cognitive dysfunction and neurotoxicity in mice (Kim et al., 2009; Zeng et al., 2021). Consistently, memory and cognitive impairment of SAMP8 mice was ameliorated by AKO, Nob, and The only to some extent in the present study. Further, AKO exhibited synergistic effects with Nob and The in ameliorating recognition memory and spatial memory deficiency in SAMP8 mice, respectively. The combination of AKO and Nob and that of AKO and The showed different synergistic effects in the Morris water maze test and in the novel object recognition test, which might be due to the different processes underlying recognition memory and spatial memory (Bevins and Besheer, 2006).

### The effects of neurofibrillary tangles and Aβ aggregation on the brain

**Figure 2:**
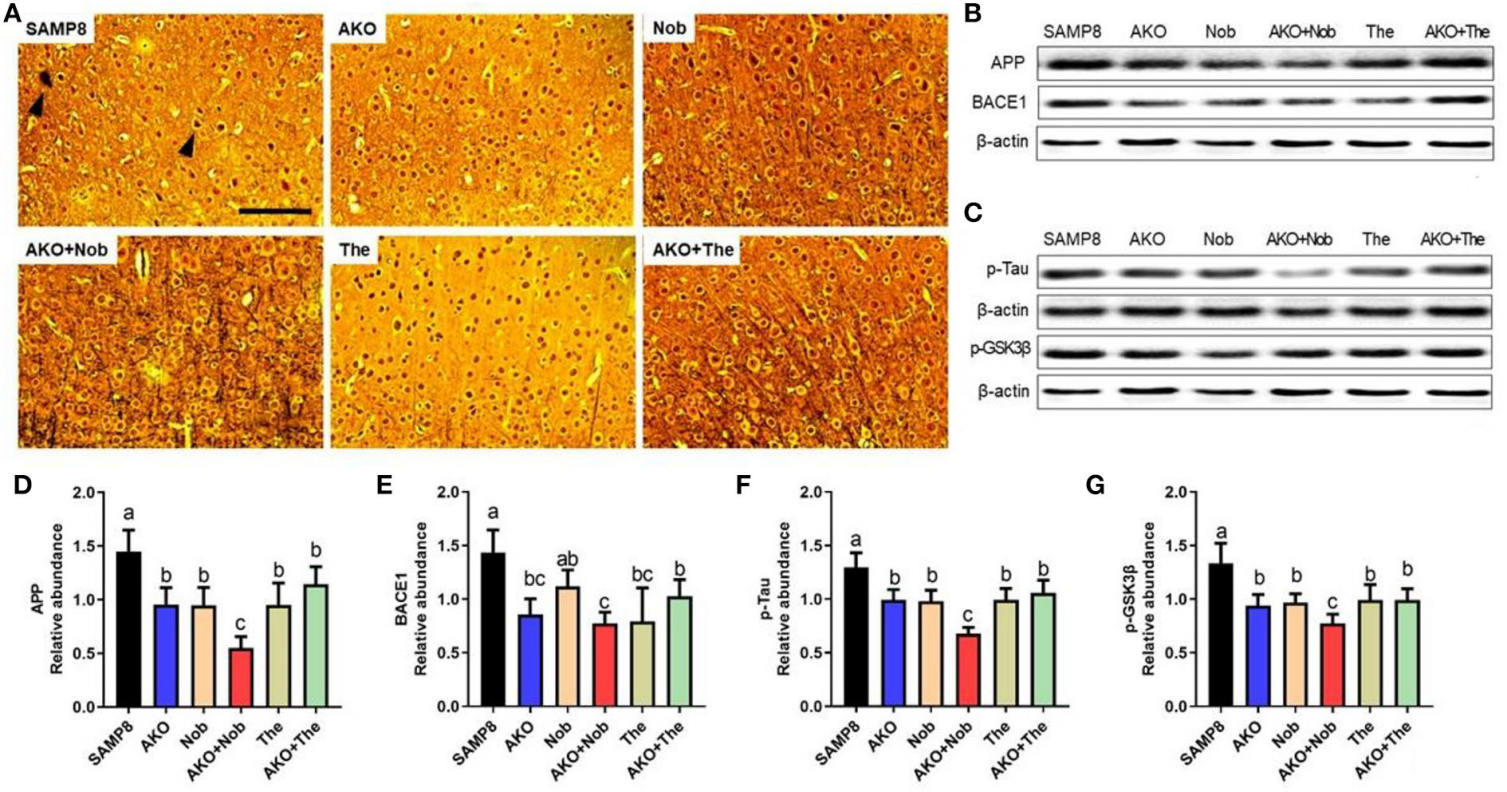
Effects of neurofibrillary tangles and β-amyloid (Aβ) aggregation. **(A)** The representative image in the brain stained by Bielschowsky silver staining, scale bar = 100 μm. Brain parenchyma contains lesions such as neurofibrillary tangles and Aβ aggregation (arrowheads). **(B)** The representative bands of amyloid precursor protein (APP) and β-site APP cleaving enzyme 1 (BACE1). **(C)** The representative bands of phosphorylated tau (p-Tau) and phosphorylated-glycogen synthase kinase-3β (p-GSK3β). **(D–G)** The relative expressions of APP **(D)**, BACE1 **(E)**, p-Tau **(F)**, and p-GSK3β **(G)**. All data were presented as mean ± SEM. Different letters indicate a significant difference at *p* <0.05 by ANOVA.

β-amyloid aggregation and neurofibrillary tangles, the main pathological characteristics of AD, were determined by Bielschowsky silver staining in the present study. The results showed that obvious Aβ plaques and neurofibrillary tangles were found in the brain of SAMP8 mice, which were alleviated to some extent by AKO+The and AKO+Nob with stronger effects than AKO, Nob, and The alone (Figure 2A). Protein levels related to Aβ aggregation and neurofibrillary tangles were subsequently determined by western blotting, and the results suggested that protein levels were significantly reduced by AKO, Nob, and The alone and in combination (Figures 2B–G). Importantly, AKO+Nob had stronger effects than others in inhibiting the expressions of proteins related to Aβ aggregation and neurofibrillary tangles, which revealed the synergistic effects of AKO with Nob in suppressing the aggregation of Aβ and neurofibrillary tangles in SAMP8 mice (Figures 2B–G). Our previous study revealed that AKO significantly suppressed the level of Aβ in the brain, and the AKO-rich n-3 PUFA-enriched phospholipids significantly inhibited the generation of Aβ by reducing the level of APP and BACE1, which was consistent with the data in the present study (Li et al., 2018; Wang et al., 2019b). In addition, it has been reported that Nob reduces intracellular and extracellular Aβ in iPS cell-derived AD model neurons and hyperphosphorylation of tau in SAMP8 mouse (Nakajima et al., 2013), and The significantly decreases the generation of Aβ in D-galactose-induced rats, which was consistent with the present study (Kimura et al., 2018; Zeng et al., 2021). Furthermore, it has been suggested in the present study that the synergistic effects of AKO with Nob rather than The reduce the generation of Aβ and neurofibrillary tangles by inhibiting APP/BACE1 and p-Tau in SAMP8 mice.

### The effects of neuronal cell loss on the brain

**Figure 3:**
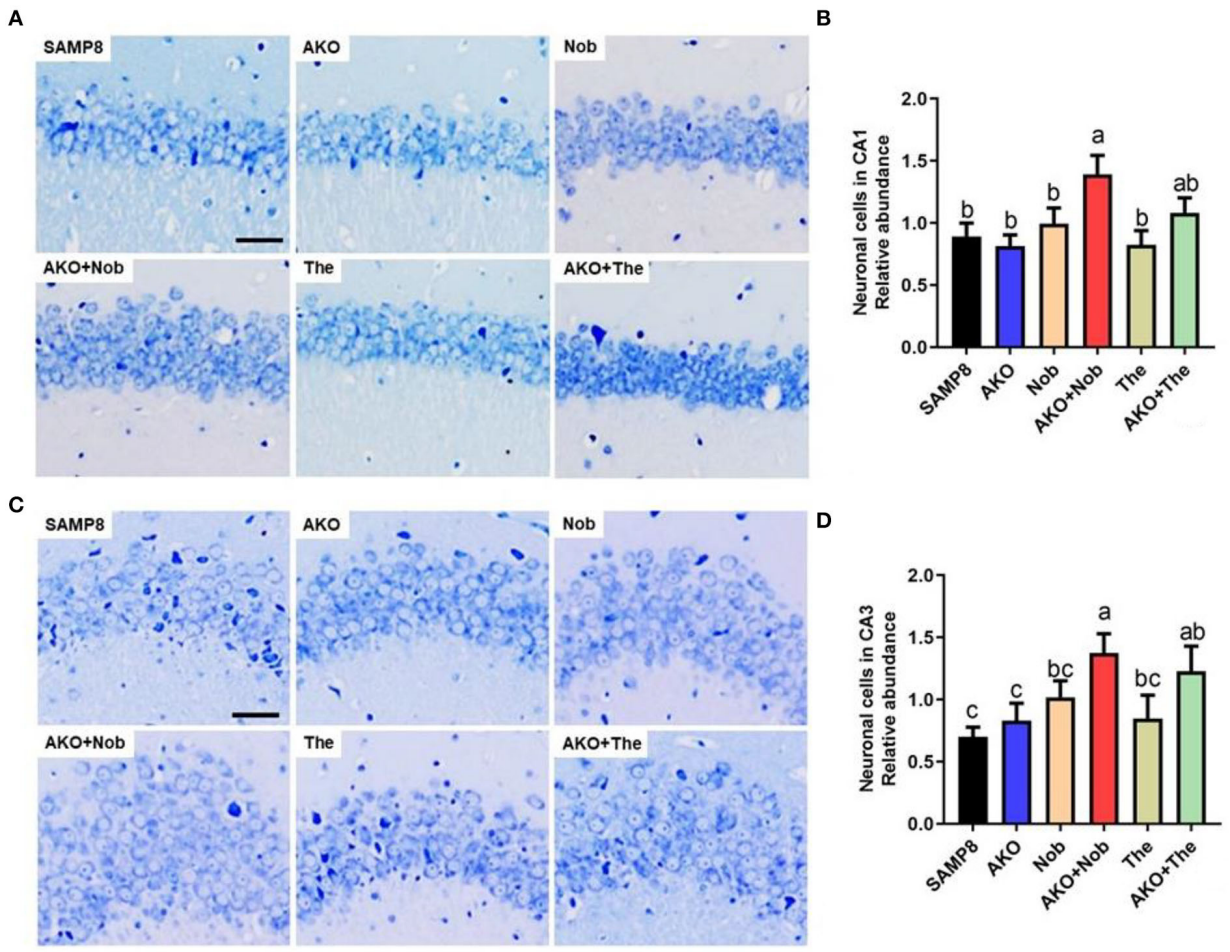
Effects of neuronal cell loss. **(A)** The representative image in CA1 of the hippocampus stained by Nissl staining, scale bar = 200 μm. **(B)** The relative number of neuronal cells in CA1 of the hippocampus. **(C)** The representative image in the CA3 of hippocampus stained by Nissl staining. **(D)** The relative number of neuronal cells in CA3 of the hippocampus. All data were presented as mean ± SEM. Different letters indicate a significant difference at *p* <0.05 by ANOVA.

Neuronal cell loss is another important pathological characteristic of AD, which is accompanied by Aβ aggregation and neurofibrillary tangles. The number of neuronal cells was determined by Nissl staining in the present study, and the results suggested that dietary AKO+Nob significantly increased the number of neuronal cells in CA1 and CA3 of the hippocampus, though no significant differences were observed in AKO and Nob alone (Figures 3A,B). In addition, the number of neuronal cells in CA3 of the hippocampus was increased by AKO+The, which was significantly superior to AKO alone (Figures 3C,D). Our previous study suggested that n-3 PUFA-enriched phospholipids significantly suppressed neuronal cell loss in Aβ-induced rats and protected SH-SY5Y cells against hydrogen peroxide-induced damage (Che et al., 2018; Wen et al., 2019). In addition, it has been reported that Nob prevents CA1 neuronal loss in Aβ1-40-induced rats (Ghasemi-Tarie et al., 2022a) and The inhibits neuronal cell loss in SAMP8 mice (Cai et al., 2018). The results revealed that AKO exhibited synergistic effects with Nob rather than The in suppressing neuronal cell loss in the brain of SAMP8 mice.

### The effects of apoptosis on the brain

**Figure 4:**
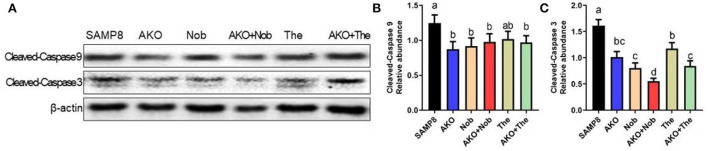
Effects of apoptosis on the brain. **(A)** The representative bands were determined by western blotting. **(B,C)** The relative expressions of Cleaved-Caspase 9 **(B)** and Cleaved-Caspase 3 **(C)**. All data were presented as mean ± SEM. Different letters indicate a significant difference at *p* <0.05 by ANOVA.

Apoptosis plays an important role in Aβ-induced neurotoxicity in AD, which leads to neuronal cell loss (Wang et al., 2019b, 2020a). In the present study, the levels of apoptosis-related proteins were determined by western blotting, and the results suggested that the level of Cleaved-Caspase 3 was significantly inhibited by AKO+Nob with stronger effects than AKO and Nob alone, and AKO+The exhibited the effects similar to AKO alone (Figures 4A,C), while the level of Cleaved-Caspase 9 was significantly suppressed by AKO+Nob, AKO, Nob, and AKO+The to the same extent (Figures 4A,B). Consistently, the anti-apoptosis effects of AKO and its n-3 PUFA-enriched phospholipids were widely reported in a variety of neurodegenerative diseases models, such as AD and Parkinson's disease (Wang et al., 2018a,b, 2019b). In addition, Nob significantly ameliorated Aβ-induced apoptosis by inhibiting Bcl-2 and Cleaved-Caspase 3 in the brain of mice (Lee et al., 2019). The above data suggested that AKO exhibited synergistic effects with Nob rather than The in suppressing neuronal cell loss by inhibiting apoptosis in the brain of SAMP8 mice.

### The effects of synaptic plasticity on the brain

**Figure 5:**
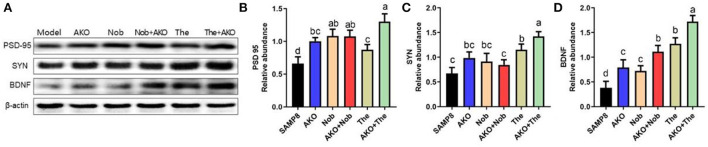
Effects of synaptic plasticity on the brain. **(A)** The representative bands were determined by western blotting. **(B–D)** The relative expressions of postsynaptic density protein-95 (PSD-95) **(B)**, synaptophysin (SYN) **(C)**, and brain-derived neurotrophic factor (BDNF) **(D)**. All data were presented as mean ± SEM. Different letters indicate a significant difference at *p* < 0.05 by ANOVA.

It has been verified that synaptic plasticity is damaged by Aβ aggregation but protected by neurotrophins, such as BDNF. The results of the present study suggested that AKO+Nob significantly increased the level of PSD-95, a typical marker of synaptic function, to the same extent with AKO and Nob alone, while AKO+The exhibited a stronger effect than AKO and The alone (Figures 5A,B). The level of SYN was increased with AKO+The with a better effect than AKO and The alone (Figures 5A,C). In addition, the level of BDNF increased with AKO+Nob with a superior effect than AKO and Nob alone, meanwhile the effects of AKO+The were stronger than those of AKO and The alone (Figures 5A,D). It has been reported that the expression of BDNF is upregulated in the hippocampus of rats receiving 7 weeks of AKO supplementation, and n-3 PUFA-enriched phospholipids significantly increased the level of SYN, which was consistent with the present study (Wibrand et al., 2013). The previous study suggested that dietary treatment with n-3 PUFA-enriched phospholipids elevated notable expressions of PSD-95 in n-3 PUFA deficient mice, contributing to recovery from cognitive deficiency (Wen et al., 2021). In addition, the expression of BDNF was also increased by Nob in rats with cerebral ischemia and chronic unpredictable mild stress-induced rats (Li et al., 2013; Zhang et al., 2013), while The significantly increased the level of BNDF in the brain of rats and mice (Wakabayashi et al., 2012; Zeng et al., 2021). It has been reported that Nob upregulates synaptic transmission *via* postsynaptic α-amino-3-hydroxy-5-methyl-D-aspartate (AMPA) receptors to restore memory impairment (Matsuzaki et al., 2008). The results in the present study suggested the synergistic effects of AKO with The in enhancing synaptic plasticity, while the synergistic effects of AKO with Nob were only observed in increasing the level of BDNF in the brain of SAMP8 mice.

### The effects of the activation of glial cells on the brain

**Figure 6:**
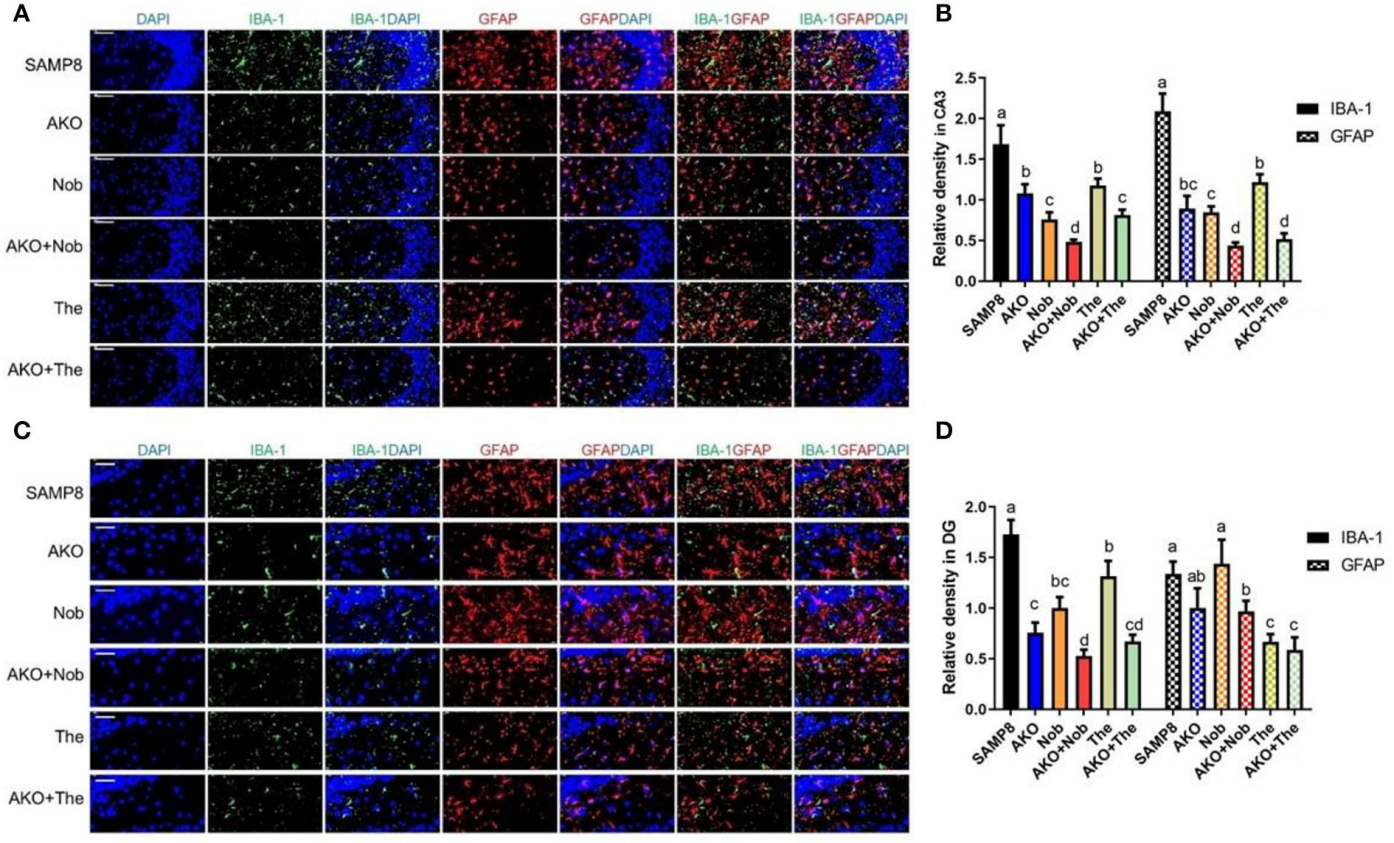
Effects on the activation of glial cells in the brain. **(A)** The representative image in CA3 of the hippocampus was determined by immunofluorescence, scale bar = 100 μm. **(B)** The relative density of ionized calcium binding adapter molecule 1 (IBA1) and glial fibrillary acidic protein (GFAP) in CA3. **(C)** The representative image in dentate gyrus (DG) of the hippocampus was determined by immunofluorescence, scale bar = 50 μm. **(D)** The relative density of IBA1 and GFAP in DG. All data were presented as mean ± SEM. Different letters indicate a significant difference at *p* < 0.05 by ANOVA.

The aggregation of Aβ can activate microglia and astrocyte, which lead to the development of neuroinflammation in the brain (Minter et al., 2016). In the present study, the activation of microglia and astrocytes in the brain was determined by their marker of activation IBA1 and GFAP, and the results showed that the levels of IBA1 and GFAP in CA3 of the hippocampus were significantly reduced by AKO+Nob and AKO+The with a stronger effect than AKO, Nob, and The alone, respectively (Figures 6A,B). In addition, IBA1 levels in dentate gyrus (DG) of the hippocampus were significantly reduced by AKO+Nob and AKO+The with a stronger effect than AKO, Nob, and The alone, respectively, while the level of GFAP was significantly reduced by AKO+Nob, AKO+The, and The alone (Figures 6C,D). It has been reported that n-3 PUFA-enriched phospholipids and Nob significantly suppress the activation of glial cells in the brain of mice, which is consistent with the present study (Wang et al., 2019b, 2021).

### The effects of neuroinflammation on the brain

**Figure 7:**
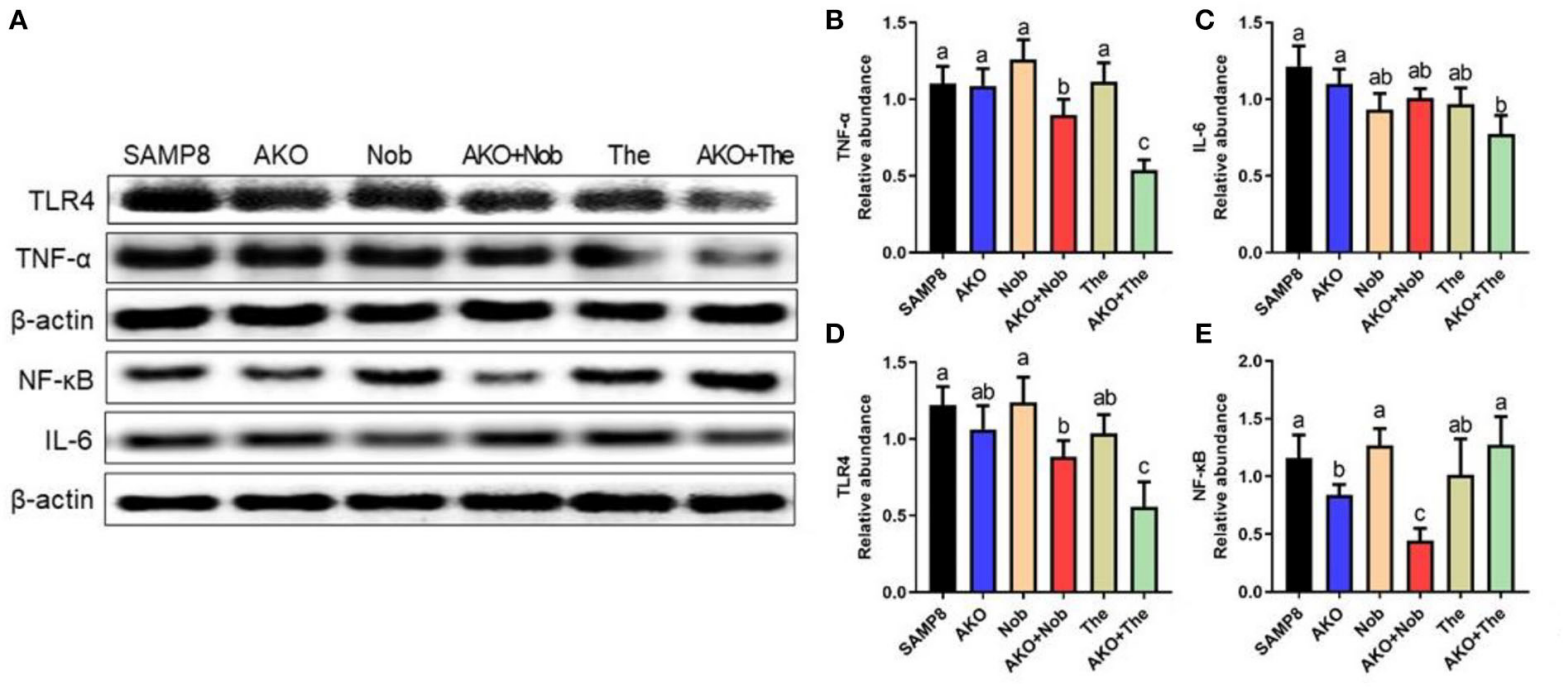
Effects of neuroinflammation in the brain. **(A)** The representative bands were determined by western blotting. **(B–E)** The relative expressions of tumor necrosis factor-α (TNF-α) **(B)**, interleukin-6 (IL-6) **(C)**, Toll-like receptor 4 (TLR4) **(D)**, and nuclear factor-κB (NF-κB) **(E)**. All data were presented as mean ± SEM. Different letters indicate a significant difference at *p* < 0.05 by ANOVA.

Further, the levels of neuroinflammation-related proteins were determined by western blotting. The results suggested that the level of TNF-α was significantly reduced by AKO+Nob and AKO+The, while AKO, Nob, and The alone exhibited no significant effects (Figures 7A,B). The level of IL-6 decreased significantly with AKO+The (Figures 7A,C). The expression and release of inflammatory factors are regulated by nuclear transcription factors NF-κB, which could be activated by TLR4. Therefore, the levels of NF-κB and TLR4 were determined and the results suggested that the level of TLR4 was significantly reduced by AKO+Nob and AKO+The, though no significant effects were found in AKO, Nob, and The alone (Figures 7A,D). In addition, the expression of NF-κB was significantly reduced by AKO+Nob with stronger effects than AKO and Nob alone (Figures 7A,E), while no significant difference was found in AKO + The groups, which revealed that NF-κB might not be involved in the synergy of AKO with The in suppressing the expression of inflammatory factors.

It has been reported that AKO protects against lipopolysaccharide- (LPS-) induced neuroinflammation (Choi et al., 2017), and n-3 PUFA-enriched phospholipids exhibits the effects of suppressing neuroinflammation in a variety of neurodegenerative diseases (Wang et al., 2019b; Du et al., 2022). In addition, the antineuroinflammatory effects of Nob and The have also been demonstrated by inhibiting LPS-induced production and secretion of proinflammatory mediators, such as TNF-α and IL-6 *in vivo* and *in vitro* (Park et al., 2018; Qi et al., 2019; Wang et al., 2019c). The abovementioned results showed that AKO exhibited synergistic effects with Nob and The in suppressing neuroinflammation in the brain of SAMP8 mice. However, the expression level of NF-kB was inconsistent with that of IL-6 and TNF-α, which might be due to other pathways involved in the expression and secretion of inflammatory factors.

**Figure 8:**
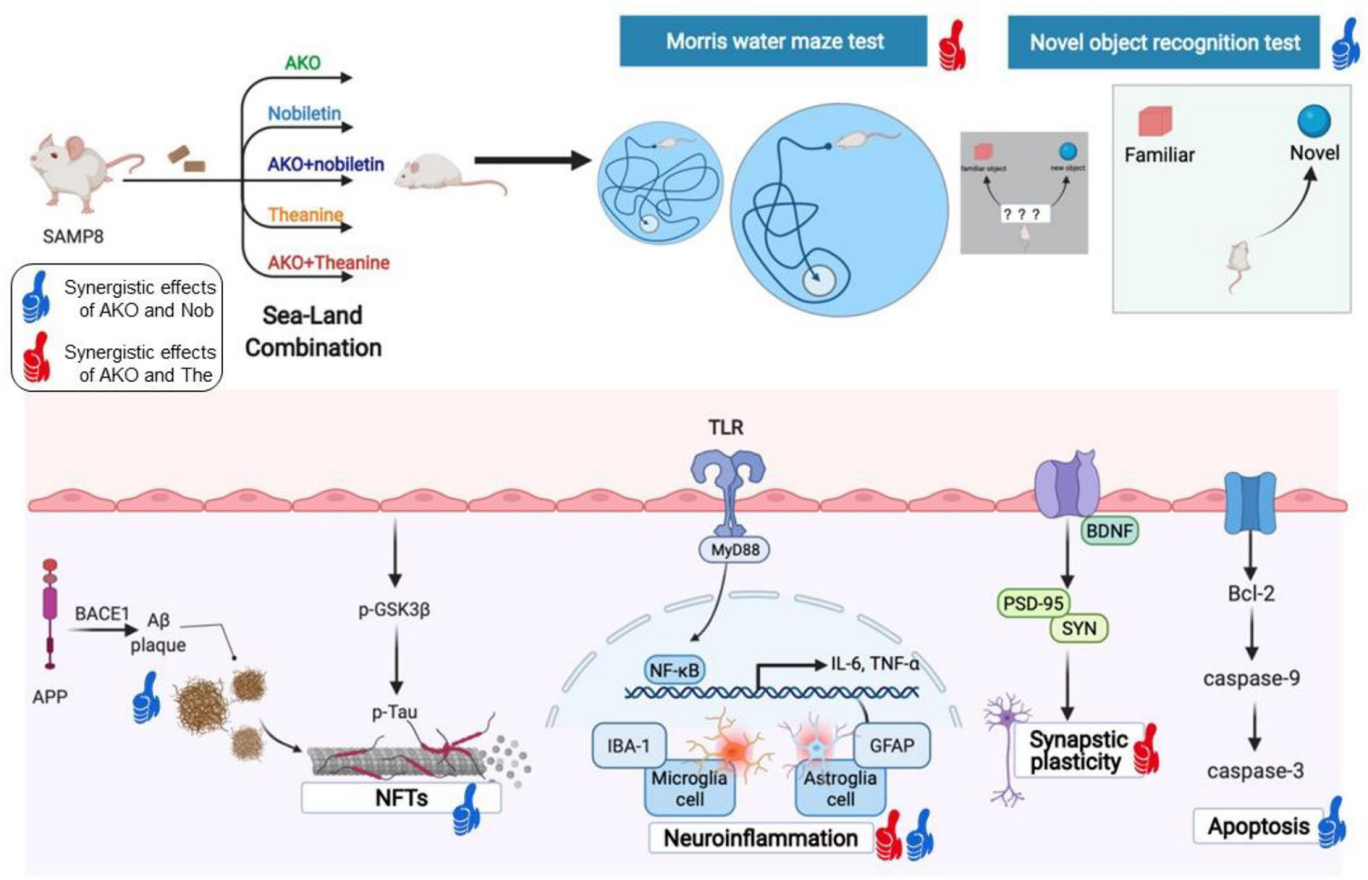
The synergistic effects of Antarctic krill oil (AKO) with nobiletin (Nob) and theanine (The) in ameliorating memory and cognitive deficiency in senescence-accelerated prone 8 mice (SAMP8) mice. The synergistic effects of AKO with Nob were signed by blue thumb, and the synergistic effects of AKO with The were signed by red thumb.

The study of mechanisms indicated the synergistic effects of AKO and Nob on Aβ aggregation, neurofibrillary tangles, apoptosis and neuroinflammation, and the synergistic effects of AKO and The on synaptic plasticity and neuroinflammation (Figure 8). The synergistic effects of AKO with Nob might be the result of a higher bioavailability of Nob. It has been reported that the soybean phospholipid increases the absorption of Nob in rats due to the increased hydrophilicity of Nob by interaction with soybean phospholipid (Lin et al., 2009). Multitarget interaction might be another important reason for the synergistic effects of AKO with Nob. It has been reported that Nob inhibits phosphodiesterase (PDE) activity and then increases the intracellular cAMP concentration to activate multiple protein kinase A (PKA) substrates (Nagase et al., 2005; Matsuzaki et al., 2008). In addition, the influx of Ca^2+^ was stimulated by activation of the N-methyl-D-aspartate (NMDA) receptors, which in turn stimulated cAMP/PKA signaling pathway and then regulated nuclear transcription factors, such as cAMP-response element binding (CREB) and NF-κB (Adams and Sweatt, 2002). In addition, AKO-rich n-3 PUFA-enriched phospholipids regulated the function of synaptic membrane-associated proteins, influencing membrane fluidity and protein–protein interactions in the brain, affecting signal transmission and synaptic function (Barceló-Coblijn et al., 2003). Another important reason for improving AD could be that n-3 PUFA-enriched phospholipids could improve brain energy metabolism and promote glucose utilization as glucose utilization was insufficient in the brain of patients with AD (An et al., 2018; Wang et al., 2019a, 2020b).

Theanine might play a complementary role with AKO through some unique mechanisms in ameliorating memory and cognitive deficiency. Due to its chemical structure similar to glutamate, The could improve neuronal function by regulating neurotransmitters, such as serotonin and dopamine (Unno et al., 1999). Furthermore, as a glutamine carrier, The inhibited the combination of extra cellular glutamine with neurons (Kakuda, 2011). The united role of sea-derived AKO and land-derived Nob and The in different aspects may be an important reason for their synergistic effects in ameliorating memory and cognitive deficiency. A broader mechanism should be involved, such as glutamatergic nerve function in the future.

## Conclusion

In summary, our study demonstrated that AKO worked synergistically with Nob in ameliorating recognition memory deficiency in the novel object recognition test and with The on spatial memory deficiency in the Morris water maze test in SAMP8 mice. Further research of the mechanism indicated that the synergistic effects of AKO and Nob in ameliorating memory and cognitive deficiency mainly involved Aβ aggregation and neurofibrillary tangles, apoptosis, while the synergistic effects of AKO and The in improving memory and cognitive deficiency mainly involved synaptic plasticity. Neuroinflammation in the brain of SAMP8 mice was synergistically inhibited by both of Nob combined with AKO and The combined with AKO. The results revealed that the sea–land combination may be an effective strategy to treat and alleviate AD, providing a perspective to retard brain aging and neurodegenerative diseases.

## Data availability statement

The raw data supporting the conclusions of this article will be made available by the authors, without undue reservation.

## Ethics statement

The animal study was reviewed and approved by Animal Ethics Committee of experimental animal care at College of Food Science and Engineering, Ocean University of China (Qingdao, China, Approval No. SPXY2020032501).

## Author contributions

Y-MW had full access to all study data and took responsibility for the integrity of the data and accuracy of the data analysis. Y-MW and C-CW conceived the original idea for the study, supervised the conception, and revised and drafted the manuscript. J-YK, X-YL, and J-YY performed behavioral tests and molecular biological analysis and analyzed the data. TY and C-HX revised the manuscript. All authors read and approved the final manuscript.

## Funding

This work is supported by National Key R&D Program of China (2018YFD0901103).

## Conflict of interest

The authors declare that the research was conducted in the absence of any commercial or financial relationships that could be construed as a potential conflict of interest.

## Publisher's note

All claims expressed in this article are solely those of the authors and do not necessarily represent those of their affiliated organizations, or those of the publisher, the editors and the reviewers. Any product that may be evaluated in this article, or claim that may be made by its manufacturer, is not guaranteed or endorsed by the publisher.
